# Healthcare policy changes in an era of health workforce shortage

**DOI:** 10.1186/s13584-023-00576-7

**Published:** 2023-08-10

**Authors:** Yoel Angel, Gil Fire

**Affiliations:** 1 Tel Aviv Sourasky Medical Center, Tel Aviv, Israel; 2 The Faculty of Medicine and the Coller School of Management, Tel Aviv University, Tel Aviv, Israel

**Keywords:** Physician shortage, Anesthesiology, Work hours reform, Foreign medical graduates, Healthcare policy, Burnout

## Abstract

In their recent IJHPR article, Wimpfheimer and colleagues outline the implications for the field of anesthesia of two major healthcare policy changes in Israel: The Yatziv Reform in licensing foreign medical graduates and the efforts to reduce residents' on-call shift duration. We argue that these reforms are necessary to strengthen the healthcare workforce and improve the quality of care in the long term, even though they may limit the availability of healthcare personnel for several years, particularly in the field of anesthesia. In this commentary, we examine the background to these policy changes, their likely impact on the medical workforce in Israel in general, and propose steps to reconcile these reforms with the global and national shortage of physicians. We urge policymakers to allocate the required resources and begin preparing for an era of continuous mismatch between physician supply and demand, which will necessitate creative solutions, increased reliance on technology, and the introduction of paramedical professionals to help offload tasks and better utilize the scarce physician workforce.

## Background

In their recent IJHPR article [1], Wimpfheimer and colleagues reviewed the status of the Israeli anesthesiology workforce and discussed the potential implications of two national medical workforce policies on the field of anesthesia: the proposed 18-h shiftwork limitation and ceasing the recognition of medical training obtained in certain foreign medical schools that do not meet quality standards. We commend the authors for their important work, which was based on a survey that achieved an impressive 100% response rate among the chairs of Israeli anesthesia departments. In this paper, we aim to examine the background to these policy changes and their impact on the medical workforce in Israel, and to suggest possible steps to reconcile these changes with the growing shortage in the medical workforce.

## The physician shortage

The shortage of healthcare personnel, particularly physicians, is a widespread global problem [2, 3]. In Israel, this shortage is particularly significant. According to data from the Organization for Economic Cooperation and Development (OECD) [4], in 2020 there were only 3.3 practicing physicians per 100,000 population in Israel, compared to the OECD average of 3.6 and the EU average of 4.0. The national shortage in physicians is expected to worsen in the coming years due to several factors: Firstly, there is an anticipated increase in the Israeli population, especially among those over the age of 65 [5]. Secondly, over 50% of Israeli physicians are over 55 years of age, the second highest share among all OECD countries [4]. Consequently, between 800 and 900 physicians are expected to retire every year during the years 2023–2027 [6], offsetting almost half of the number of new physicians that enter the system. Internal data from the Israeli Ministry of Health (MoH) indicate that in approximately a decade, this may disproportionally affect anesthesia services, as there is a higher proportion of anesthesiologists nearing retirement compared to other specialties (Alexey Belinski, Israeli MoH, personal communication). Lastly, there is a growing demand for more physicians to reduce wait times, improve quality of care and provide better-specialized care in many fields of medicine. For example, the 2019 Tur-Caspa report [7] emphasized the need for a higher number of physicians in Internal Medical wards. Yet another example is a survey conducted in 2021, indicating that one third of patients who seeked an outpatient specialist appointment had to wait more than 30 days, which likely reflects a shortage in specialists in other fields of medicine as well [8].

Compounding the shortage, Israel has a low number of annual medical graduates, with only 7.2 annual graduates per 100,000 population, nearly half of the OECD average of 13.5. Furthermore, Israel has the highest proportion of foreign-trained physicians among OECD countries, with approximately 58% of physicians trained abroad compared to the OECD average of 18% [4]. Lastly, Israel's national expenditure on health is 7.5% of its GDP, which is lower than the OECD average of 8.8% [4]; the share of public spending from total health expenditure is 65%, also lower than the OECD average. These data suggest there are limited available resources for increasing the number of physician positions or incentivizing physicians to fill less sought-after roles in the public system, although public spending is only one of many factors influencing physician choice of employment.

In addition to the national physician shortage, there are significant disparities in healthcare resources between the central and peripheral regions of Israel and between medical specialties. The periphery has fewer healthcare personnel and a higher percentage of foreign-trained physicians. Some specialties, like family medicine or plastic surgery, enjoy a consistently high number of applicants, while the number of residency candidates for other less sought-after specialties like psychiatry, geriatric medicine, neonatal intensive care, and anesthesia is more sensitive to the total number of available applicants, especially in the periphery. The Israeli MoH has only recently started monitoring and planning the health workforce and is now taking several steps to address this shortage. These include installment of a permanent body for long term health workforce planning; increasing the annual number of domestic first year medical students by 71% by 2024 and further increasing it thereafter; incentivizing Israeli medical students abroad to return to Israel at the end of their training; facilitating the migration of foreign-trained physicians to Israel; encouraging doctors to train and practice in the periphery; and several other steps [6]. At the commission of the Israeli MoH, the OECD has also recently issued a comprehensive report addressing the status of the physician workforce in Israel [9], and its recommendations are currently being considered. Should all these steps materialize, Israel will close the gap and enjoy a stable supply of well-trained physicians. However, this is only expected to happen within a decade or so.

Yet, despite the need to increase the number of physicians across all specialties and regions, the MoH is also championing two major policy changes that may reduce the availability of the medical workforce in the near future. It is crucial to understand the motivations behind these steps.

## The Yatziv reform in licensing of foreign medical graduates

In 2019, the Israeli MoH initiated an investigation into the quality of training provided by foreign medical schools due to reports of inadequate medical competence among some foreign-trained physicians. The investigation, led by Prof. Shaul Yatziv of the Israeli MoH, revealed that certain schools did not meet academic standards and did not provide sufficient clinical exposure.

As a result, a reform was implemented in the licensing process, preventing students who enrolled from 2019 onwards to these medical schools, mostly in non-OECD countries, from obtaining an Israeli medical license. As medical school lasts 6 years, the effects of this policy will reach the Israeli healthcare system starting in 2025. These effects are unavoidable at this point, as no Israeli students have enrolled to these schools in the past 5 years. According to MoH data, approximately 60% of foreign-trained physicians (or 34% of all new physicians) who received their license in 2022 had graduated from medical schools that will no longer be recognized under the Yatziv reform [6].

This reform will significantly decrease the annual number of new physicians in the short term, but will reduce the reliance on foreign medical schools in the long run. Notably, the MoH data mentioned in the article by Wimpfheimer et al. highlights that anesthesia departments, especially those located in the outskirts of Israel, have a larger share of physicians who trained at these medical schools compared to other departments, and will therefore be more significantly impacted by the reform. Nevertheless, we consider this decision to prioritize physicians' competence and professionalism over sheer numbers to be commendable and courageous.

## Reduction of overnight shift duration

For over a decade, there has been a growing voice among young physicians in Israel, mostly in non-surgical specialties, calling for a shorter duration of on-call shifts, currently set at 26 h. In recent years, there have been protests, strikes and other work actions, and even calls for mass resignation by hundreds of doctors advocating for this reform. In 2021, following an Executive Order by the Secretary of Labor, a national committee was established to plan the implementation of this reform. However, the committee failed to reach a consensus among its members on how, or even if, such a change was possible in the foreseeable future [10]. The MoH supported a gradual implementation of 16-h shifts in non-surgical wards, while several other stakeholders believed that no reduction was possible due to the national shortage of physicians discussed earlier. Ongoing discussions now consider an alternative of 21-h shifts. It should be emphasized that anesthesia wards were never planned to be included in the first stage of the reform due to existing personnel shortages and the high burden of on-call shifts in these wards, which would require an unfeasible number of additional anesthesiologists to facilitate shorter shifts without compromising the level of service. Surgical wards were also excluded from the first stage due to similar concerns. Therefore, contrary to what might be inferred from reading the original paper by Wimpfheimer et al., plans to implement shorter shifts in anesthesia are still in very preliminary stages.

Efforts to shorten work hours are also taking place in other countries [11, 12]. While the mechanism and roadmap for reducing work hours remain a matter of debate, there is a growing consensus on its necessity. Long work hours are associated with increased burnout [13], a major concern for healthcare organizations worldwide. Many members of the current generation of physicians have expressed their unwillingness to tolerate the existing working conditions for long, and some may consider alternative career paths, such as residencies that do not include shift work or leaving the public health system, or even the practice of medicine, altogether.

These effects of existing work schedules severely threaten the Israeli health system's ability to retain current staff and attract new members. This threat will not disappear on its own; ignoring it will only delay the inevitable crisis rather than resolve it. Moreover, it is reasonable to assume that if, and when, some fields in medicine introduce a shortened shift schedule, it will potentially lead to a shift of physicians away from specialties that maintain a prolonged shift schedule.

For these reasons, we believe implementation of a more balanced work schedule in anesthesia is of upmost importance. We agree with the authors that shortening the work hours of anesthesiologists is not currently feasible. Nonetheless, steps must be taken immediately to establish an infrastructure so that change can be implemented in the future.

We also acknowledge other concerns associated with shortening on-call shifts, such as a potential negative effect on the quality of clinical training or an increase in handoffs that may adversely affect patient safety. These are serious concerns that should be addressed. Measures to mitigate such risks should be taken, including revising residency programs, transitioning to competency-based training, and implementing mechanisms and procedures to enhance patient safety. We do not believe, however, that these concerns should serve as reasons to abstain from necessary reforms.

Lastly, Wimpfheimer and colleagues state that "it is not clear what the net benefits will be in terms of patient safety" and indeed, studies examining the effects of shortening work hours on patient safety yield conflicting results [14–17]. However, we must bear in mind that the motivation for this change is not only the commitment to patients but also to the safety and well-being of the physicians treating these patients.

## The challenges unique to anesthesia

The authors report that despite a consistent rise in the demand for anesthesia residencies over the past decade, “anesthesiology remains an unpopular career choice among Israeli medical graduates”. This situation can be seen as a chicken-and-egg problem: the existing shortage of anesthesiologists contributes to anesthesia's unpopularity as a career choice, which in turn worsens the shortage.

Steps must be taken to understand the underlying reasons for this trend and rectify them. While some possible causes are inherent to the profession, such as the nature of clinical work or the absence of long-term relationships with patients, other factors can be modified, including working conditions, compensation, shift duration, motivation and teaching approach of senior staff, and the clinical and academic prestige associated with anesthesia in Israel.

One possible explanation for the high percentage of anesthesiologists trained in non-accredited medical schools is that anesthesia became a "last resort" for applicants who had limited career alternatives. If this is the case, the scarcity of Israeli medical graduates choosing to specialize in anesthesiology perpetuates a vicious cycle that discourages others from pursuing it.

The Yatziv reform could be one of the factors that break this cycle: increasing the proportion of Israeli-trained physicians in the field may encourage more Israeli-trained physicians to consider anesthesiology as a specialty. Additionally, reducing workload and shift duration may make anesthesiology more attractive to young physicians who prioritize achieving a work-life balance. Lastly, in addition to the shortage of residents, many anesthesia wards also suffer from a lack of senior physicians. Attracting additional specialists and retaining the existing ones is crucial and requires many steps and policy changes, but discussing them is beyond the scope of this commentary.

## Facing the challenges ahead

As the shortage of physicians is expected to worsen in the future, and advancements in medicine will continue to increase the demand for physicians across all specialties, this supply–demand imbalance is likely to continue and affect healthcare systems worldwide. While this should be seen as a constraint, it should not discourage healthcare leaders from making changes necessary to ensure optimal delivery of care and the safety and well-being of healthcare workers. Instead, it should motivate them to seek new and creative solutions.

Since training new physicians takes many years, fast solutions are needed. These may include offering financial and other incentives to attract physicians to areas of need (similar incentives were among the reasons for an increase in the number of anesthesiologists in the previous decade), re-hiring retired physicians seeking employment, and developing novel work models to utilize existing personnel better. It should be noted that financial incentives to specialties of need are not a sustainable solution, considering the shortage exists in many specialties. Offering higher wages for physicians in one field will reduce the number of physicians in other fields, and over time will drive up the spending on physician salaries across all specialties.

Hopefully, these steps will adequately address the workforce shortage during the interim period until the number of physicians is restored to its current standards. Still, the MoH will have to diligently monitor service availability across different fields to ensure this objective is achieved. If there are instances where service availability reaches a critical level, the Israeli government may have to consider additional measures to ensure the population's healthcare needs are met, in line with its responsibility according to the Israeli National Health Insurance. This could involve procuring services from the domestic private health sector and, in extreme cases, even seeking assistance from foreign providers.

In the long term, all medical fields, particularly anesthesia, must find ways to do more with less. This could involve relying more on promising technological advancements such as closed-loop anesthesia delivery systems [18], remote monitoring, and artificial intelligence decision support systems [19]; drugs can be prepared in the hospital pharmacy to conserve physician time, and digital systems may replace or minimize preoperative visits for some patients.

Additionally, para-medical personnel like Physician Assistants (PAs) or nurse anesthetists can help with time-consuming tasks of a technical nature such as operating room preparation, insertion of IV lines and premedication. The medical establishment in Israel has traditionally opposed the introduction of physician assistants to various fields, including anesthesia, due to concerns about care quality, clinical exposure for trainee doctors, professional prestige, and physician income. These concerns are not unfounded, and evidence from other countries that have introduced physician assistants into the operating room is conflicting [20, 21]. Israel is not the only country where professional societies oppose the introduction of the para-medical personnel into the field of anesthesia [22]. Nonetheless, we believe that with careful planning and clear definition of responsibilities, integrating para-medical personnel into the operating room workflow can allow physicians to focus on the most cognitively demanding and non-technical tasks, while also providing better training for residents, without compromising the unique role of physicians in patient care. Such a shift has been previously proposed as a major step in reducing physician burnout [23]. The Israeli MoH is actively promoting legislation to introduce a new generation of PAs who will undergo a comprehensive two-year graduate training program. These highly skilled PAs will enjoy significantly greater privileges than para-medical personnel previously had in Israel. They will be integrated into various fields of medicine, provided that the relevant medical societies choose to embrace their inclusion. We are optimistic that anesthesia will be one of the fields where this exceptional opportunity will come to fruition.

We agree with the authors' conclusion that long-term planning by regulatory authorities must consider input from those working in the field, and that reforms should optimally take place only after thorough risk analysis and preparation. In the case of the Yatziv reform, such prospective planning could have eliminated some of the implementation problems currently encountered. However, it must be acknowledged that political and organizational circumstances often limit the ability to follow such a comprehensive process. The structure of the Israeli government, specifically the Ministry of Finance’s near-exclusive control of budget allocation to other government offices` initiatives, makes such a process extremely difficult. Facing a dilemma of either pursuing a reform and managing the resulting consequences or abandoning the reform altogether, we commend decisionmakers for setting ambitious goals and taking bold steps in the face of uncertainty.

Lastly, we also acknowledge that true reform requires the proper allocation of resources. Increasing the national expenditure on health from public sources to bring it to par with other OECD countries is a vital policy step in this regard.

## Conclusion

The reform in physician’s licensing in Israel is underway and will soon take effect, and the long-awaited reform in work hours restrictions is expected to begin implementation, although the timeline for its implementation in anesthesia remains uncertain. Some reduction in the availability of healthcare personnel over the coming years, particularly in anesthesia, is expected. Nonetheless, both these reforms are essential to strengthen health workforce and enhance quality in the medium and long term. The Yatziv reform will improve the professional standards of anesthesia residents and make it a more appealing career choice for young, motivated physicians. Implementing a shorter shift schedule may incentivize more physicians to specialize in anesthesia and help retain the current staff. To minimize any potential negative impact on service delivery resulting from these reforms, the Israeli healthcare system must begin preparations promptly. For this to happen, the Israeli government and parliament must allocate sufficient resources, and leaders—from high-ranking decisionmakers to department chairs—must work proactively and in collaboration to facilitate the necessary changes.

## Data Availability

Not applicable.
